# Individual classification of ADHD patients by integrating multiscale neuroimaging markers and advanced pattern recognition techniques

**DOI:** 10.3389/fnsys.2012.00058

**Published:** 2012-08-06

**Authors:** Wei Cheng, Xiaoxi Ji, Jie Zhang, Jianfeng Feng

**Affiliations:** ^1^Centre for Computational Systems Biology, Fudan UniversityShanghai, P.R. China; ^2^Mathematical Department, Zhejiang Normal University, JinhuaZhejiang Province, P.R. China; ^3^Department of Computer Science, Warwick UniversityCoventry, UK

**Keywords:** ADHD, functional brain networks, pattern classification, fALFF, ReHo, BWAS

## Abstract

Accurate classification or prediction of the brain state across individual subject, i.e., healthy, or with brain disorders, is generally a more difficult task than merely finding group differences. The former must be approached with highly informative and sensitive biomarkers as well as effective pattern classification/feature selection approaches. In this paper, we propose a systematic methodology to discriminate attention deficit hyperactivity disorder (ADHD) patients from healthy controls on the individual level. Multiple neuroimaging markers that are proved to be sensitive features are identified, which include multiscale characteristics extracted from blood oxygenation level dependent (BOLD) signals, such as regional homogeneity (ReHo) and amplitude of low-frequency fluctuations. Functional connectivity derived from Pearson, partial, and spatial correlation is also utilized to reflect the abnormal patterns of functional integration, or, dysconnectivity syndromes in the brain. These neuroimaging markers are calculated on either voxel or regional level. Advanced feature selection approach is then designed, including a brain-wise association study (BWAS). Using identified features and proper feature integration, a support vector machine (SVM) classifier can achieve a cross-validated classification accuracy of 76.15% across individuals from a large dataset consisting of 141 healthy controls and 98 ADHD patients, with the sensitivity being 63.27% and the specificity being 85.11%. Our results show that the most discriminative features for classification are primarily associated with the frontal and cerebellar regions. The proposed methodology is expected to improve clinical diagnosis and evaluation of treatment for ADHD patient, and to have wider applications in diagnosis of general neuropsychiatric disorders.

## Introduction

Attention deficit hyperactivity disorder (ADHD) is characterized by clinical symptoms of inattention, impulsivity, and hyperactivity. It is one of the most common brain and behavioral disorders among children, which affects 5–8% school age children. ADHD can frequently persist into adolescence and adulthood (Biederman, 2004; Barkley, 2006), which can cause significant functional impairments in the brain (Frances, 1994). A number of neuroimaging studies have demonstrated the abnormalities in both structure and function of the brain for ADHD patients (Seidman et al., 2005; Bassett et al., 2006). Structural abnormalities involve reduced volume and cortical thickness found in frontal, parieto-temporal, cingulate regions, cerebellum, and corpus callosum (Krain and Castellanos, 2006; Shaw et al., 2006; Mackie et al., 2007; Carmona et al., 2009; Batty et al., 2010; Rubia, 2011). Functional connectivity alterations of ADHD patients include fronto-parietal (Dickstein et al., 2006), fronto-striatal (Castellanos et al., 2006), and frontotemporal-parietal network (Smith et al., 2006), and also anterior cingulate (Tian et al., 2006).

Although there have been extensive studies of ADHD in terms of widespread brain regions and the connectivity patterns, relatively less attention are focused on the pattern classification based on the neuroimaging data of individual ADHD patients, which is crucial for subjective and accurate clinical diagnosis of ADHD (Zhu et al., 2008). Compared with identifying differences at the group level, pattern classification on the individual level proves to be a more difficult task. It should be approached with highly sensitive neuroimaging markers, and efficient feature-selection/pattern recognition approaches (Zhang et al., 2012). As a specific example, consider the hippocampal volume measurements for individuals in two samples. Suppose a two sample *t*-test comparison of the two samples resulted in a significantly small *p*-value. Generally, it will be hard to accurately distinguish (e.g., with 90% accuracy) which sample an individual is drawn from, because the hippocampal volume of these two samples may have substantially overlapping distributions. In other words, finding group difference only requires a *p* value that is less than a threshold, while accurately distinguishing the two samples, at the individual level, requires that the two samples to be substantially separated, which requires a highly significant *p* value. Thus only highly discriminative features (having extremely significant *p* value) can lead to a good performance in classification.

Despite the success in classifying various other brain disorders such as schizophrenia, Alzheimer's disease, depression, epilepsy etc. (Hahn et al., 2011; Liu et al., 2011; Zhang et al., 2011a,b), the work on classification of ADHD remains limited. Moreover, most results in the literature are based upon a small population of patients (in the order of tens) and the obtained results cannot be fully validated, and hence their clinical significance is still limited.

In this paper, we address the problem of accurately classifying the brain state (healthy or with ADHD disorders) on an individual basis for a large data set. In particular, we will summarize the neuroimaging features that are highly discriminative across the healthy group and the ADHD patients, which include both local measures such as fractional amplitude of low frequency fluctuations (fALFF) (Zou et al., 2008) and regional homogeneity (ReHo) (Zang et al., 2004), and the global characteristics like the functional connectivity derived from various definitions. A brain-wide association study (BWAS) (Ji et al., 2012) and feature integration are performed to extract the most sensitive features, which we found to be closely associated with the frontal and cerebellar regions. Finally, the correlation between these neuroimaging markers and the ADHD index is presented.

## Materials and methods

### Participants and data acquisition

The fMRI data used in this paper are from the ADHD-200 Consortium for the global competition (http://fcon_1000.projects.nitrc.org/indi/adhd200/). Since the fMRI data collected from different centers may have some systematic differences that are possibly caused by the fMRI machine used, in this paper we only use the fMRI data collected from the Institute of Mental Health and National Key Laboratory of Cognitive Neuroscience and Learning (Peking University, Beijing, China) to minimize variability across institutions. There are 244 children, 143 of which are healthy controls (59 females, 84 males; mean age 11.43 ± 1.86 years; mean index 29.34 ± 6.41), and the rest 101 are patients with ADHD (10 females, 91 males; mean age 12.08 ± 2.05 years; mean index 50.43 ± 8.42), including 38 ADHD-Combined (ADHD-C) and 63 ADHD-Inattentive patients (ADHD-I).

All participants (ADHD and controls) are evaluated by the Schedule of Affective Disorders and Schizophrenia for Children—Present and Lifetime Version (KSADS-PL) with one parent for the establishment of the diagnosis. The ADHD Rating Scale (ADHD-RS) IV is employed to provide dimensional measures of ADHD symptoms. All subjects are assessed for intelligence quotients (IQ) on the Wechsler Intelligence Scale for Chinese Children-Revised (WISCC-R) (mean IQ score 113 ± 14.40), and for ADHD index on the 18-item version of the ADHD-RS IV (mean index: 38.31 ± 12.76). Additional inclusion criteria include: (1) right-handedness, (2) no lifetime history of head trauma with loss of consciousness, (3) no history of neurological disease and no diagnosis of schizophrenia, affective disorder, pervasive development disorder, and substance abuse, and (4) a full scale (WISCC-R) score greater than 80.

Five subjects (two healthy controls and three ADHD patients) showed large head movements (exceeding 3 mm translation or 3° rotation) and thus are excluded from our analysis.

### Data processing

All functional imaging data are acquired using an acronym for Analysis of Functional NeuroImages (AFNI) and FSL (http://www.fmrib.ox.ac.uk/fsl/). Before functional image preprocessing, the first four volumes are discarded to allow for scanner stabilization. Briefly, the remaining functional scans are first corrected for within-scan acquisition time differences between slices, and are then realigned to the middle volume to correct for inter-scan head motions. After this the functional scans are spatially normalized to a standard template (Montreal Neurological Institute) and resampled to 4 mm × 4 mm × 4 mm voxel resolution. After normalization, the Blood Oxygenation Level Dependent (BOLD) signal of each voxel is first detrended to abandon linear trend and then passed through a bandpass filter (0.009 Hz < f < 0.08 Hz) to reduce low-frequency drift and high-frequency physiological noise. Finally, nuisance covariates including head motion parameters, global mean signals, white matter signals, and cerebrospinal fluid signals are regressed out. An automated anatomical labeling (AAL) atlas (Tzourio-Mazoyer et al., 2002) was used to parcellate the brain into 90 regions of interest (ROIs) (45 in each hemisphere). The names of the ROIs and their corresponding abbreviations are listed in Table 1. We hereby appreciate what they, Carlton Chu, Virginia Tech's ARC, the ADHD-200 consortium, and the Neuro Bureau (http://neurobureau.projects.nitrc.org/NeuroBureau/Welcome.html), have done for us.

**Table 1:** **The names and abbreviations of the regions of interest (ROIs)**.

**Regions**	**Abbr.**	**Regions**	**Abbr.**
Amygdala	AMYG	Orbitofrontal cortex (middle)	ORBmid
Angular gyrus	ANG	Orbitofrontal cortex (superior)	ORBsup
Anterior cingulate gyrus	ACG	Pallidum	PAL
Calcarine cortex	CAL	Paracentral lobule	PCL
Caudate	CAU	Parahippocampal gyrus	PHG
Cuneus	CUN	Postcentral gyrus	PoCG
Fusiform gyrus	FFG	Posterior cingulate gyrus	PCG
Heschl gyrus	HES	Precentral gyrus	PreCG
Hippocampus	HIP	Precuneus	PCUN
Inferior occipital gyrus	IOG	Putamen	PUT
Inferior frontal gyrus (opercula)	IFGoperc	Rectus gyrus	REC
Inferior frontal gyrus (triangular)	IFGtriang	Rolandic operculum	ROL
Inferior parietal lobule	IPL	Superior occipital gyrus	SOG
Inferior temporal gyrus	ITG	Superior frontal gyrus (dorsal)	SFGdor
Insula	INS	Superior frontal gyrus (medial)	SFGmed
Lingual gyrus	LING	Superior parietal gyrus	SPG
Middle cingulate gyrus	MCG	Superior temporal gyrus	STG
Middle occipital gyrus	MOG	Supplementary motor area	SMA
Middle frontal gyrus	MFG	Supramarginal gyrus	SMG
Middle temporal gyrus	MTG	Temporal pole (middle)	TPOmid
Olfactory	OLF	Temporal pole (superior)	TPOsup
Orbitofrontal cortex (inferior)	ORBinf	Thalamus	THA
Orbitofrontal cortex (medial)	ORBmed		

### Neuroimaging features

#### Local features from the functional brain network

***Fractional amplitude of low-frequency fluctuation (fALFF).*** Amplitude of low-frequency fluctuation (ALFF) measures the magnitude of the fluctuation of the voxel (Zang et al., 2007). It reflects the “energy” of the BOLD signal at each voxel, which is calculated from the power spectrum of the BOLD time series. fALFF is the ALFF of a given frequency band expressed as a fraction of the sum of amplitudes across the entire frequency range in a given signal (Zou et al., 2008), i.e., the ratio of the power spectrum of low-frequency (0.009–0.08 Hz) to that of the entire frequency range, which represents the relative contribution of specific low frequency oscillations to the whole detectable frequency range.

***Regional Homogeneity (ReHo).*** It is assumed that for a given voxel, its activity is usually correlated to that of its neighbors, and ReHo is used to characterize the degree of local synchronization of spontaneous fMRI signals (e.g., within a cluster) by calculating the Kendall coefficient of concordance (KCC) (Kendall and Gibbons, 1990). KCC is defined in a voxel-wise manner as follows (Zang et al., 2004):

$$W=\frac{\sum_{i=1}^{n}{\left(R_{i}\right)}^{2}-n{\left(\bar{R}\right)}^{2}}{\frac{1}{12}K^{2}(n^{3}-n)}$$

where *W* is the KCC value of a voxel; *n* is the number of time points (here *n* = 231); *R*_*i*_ is the sum rank of all *K* voxels at the *i*th time point; $\bar{R}=\frac{K(n+1)}{2}$ is the mean of the *R*_*i*_'s; *K* is the number of selected neighboring voxels. Here, we select a given voxel together with its nearest 26 neighbors, that is, *K* = 27.

#### Global features from the functional brain network

It has been suggested that many functional brain disease like Alzheimer's disease, schizophrenia, and autism can be described as dysconnectivity syndromes, which is related to the disruption of the connectivity patterns among the spatially distributed brain regions that underlie the normal functioning of the brain (Sporns, 2011). The following features are derived from the functional connectivity by various definitions, for example, Pearson, partial and spatial correlation.

***Functional brain network by Pearson correlation.*** The most frequently used functional connectivity measure is the Pearson correlations between regional BOLD time series, which characterizes the synchronization of the regional activity in terms of the low frequency fluctuation. Here the Pearson correlation we use is based on a parcellation of the cortex into 351 brain regions, i.e., CC400 atlases in the competition website (http://www.nitrc.org/plugins/mwiki/index.php/neurobureau:AthenaPipeline) and the Pearson correlation coefficient between regional BOLD signals is computed as

$$r_{ij}=\frac{\sum_{t=1}^{T}\left[x_{i}(t)-{\bar{x}}_{i}\right]\cdot \left[x_{j}(t)-{\bar{x}}_{j}\right]}{\sqrt{\sum_{t=1}^{T}{\left[x_{i}(t)-{\bar{x}}_{i}\right]}^{2}}\cdot \sqrt{\sum_{t=1}^{T}{\left[x_{j}(t)-{\bar{x}}_{j}\right]}^{2}}}$$

where *x*_*i*_(*t*) and *x*_*j*_(*t*) (*t* = 1, 2, …, *T*, *T* = 231) are the regional time courses of region *i* and *j* with means ${\bar{x}}_{i}$ and ${\bar{x}}_{j}$, respectively. A Fisher's *r*-to-*z* transform is utilized to convert each correlation coefficient to satisfy the assumption of normality. The resultant functional brain network is 351^*^351, from which we shall extract the most sensitive features (i.e., links in the network) that are used later in the classification.

***Functional brain network by partial correlation.*** The functional connectivity revealed by Pearson correlation may not reflect the true interaction between a pair of brain regions, as it does not eliminate the effect from other brain regions that may exert influence to a pair of brain regions in question (Tao et al., 2011). Here we use partial correlation analysis as a way to reflect the true statistical dependencies between two regions after removing the confounding effects of all other regions.

***Functional brain networks at the spatial scale.*** Another kind of functional connectivity is the spatial connectivity, which is the similarity of the region-based correlation maps measured by computing the spatial correlation coefficient (Fox et al., 2006; Vincent et al., 2007). It is similar to the Pearson correlation, but instead of computing the correlation across time points, it computes the correlation across regions. Thus, for correlation maps corresponding to region *i* and *j*,

$$R_{ij}=\frac{\sum_{n=1,n\neq i,n\neq j}^{N}\left[z_{i}(n)-{\bar{z}}_{i}\right]\cdot \left[z_{j}(n)-{\bar{z}}_{j}\right]}{\sqrt{\sum_{n=1,n\neq i,n\neq j}^{N}{\left[z_{i}(n)-{\bar{z}}_{i}\right]}^{2}}\sqrt{\sum_{n=1,n\neq i,n\neq j}^{N}{\left[z_{j}(n)-{\bar{z}}_{j}\right]}^{2}}}$$

where *z*_*i*_(*n*) and *z*_*j*_(*n*)(*n* = 1, 2…*N*, *n* ≠ *i*, *n* ≠ *j*, *N* = 90,) are the *i*th and *j*th columns of the Pearson correlation matrix obtained above (after Fisher's transformation) with means being ${\bar{z}}_{i}$ and ${\bar{z}}_{j}$, respectively. The spatial correlation coefficient between two brain regions represents the degree of similarity in the global functional connectivity patterns of the two regions.

### Pattern classification

#### Feature selection

In the above section, we have listed all the features that we have used and all of them are high-dimensional by nature. For example, CC400 atlases network contains 61425 (351 × 350/2) links among different regions of interest (ROIs). For voxel-level features such as ReHo and fALFF, the corresponding dimensionality can be even higher since they are measured at the voxel level. This high dimensionality in features can lead to the “curse of dimensionality” problem and greatly hamper the performance of the classifier. To reduce the dimensionality of the feature space, on one hand, two-sample two tailed *t*-tests are performed to select the sensitive features from fALFF, ReHo, Pearson correlation, and spatial correlation (CC400 atlases), which show significant differences between the ADHD and healthy control groups; On the other hand, a BWAS is performed to select the features from the functional network derived from partial correlation. In both procedures, statistically significant features (*p*-value of two-sample *t*-test being smaller than a threshold) are selected. Note that in each leave-one-out cross-validation (LOOCV) fold, we perform *t*-tests only on the training samples to select the discriminative features.

***BWAS approach.*** Since the distribution of partial correlation is generally not Gaussian, traditional two-sample *z*-test cannot be applied directly. Here we use a BWAS approach to select the significantly altered functional connections (Ji et al., 2012). We assume there is one group of subjects suffering from ADHD, and another group of matched healthy controls. Denote these two groups as *P* and *H* respectively and the total numbers of subjects are *N*_*P*_ and *N*_*H*_. We further assume that the whole brain is parcellated into *N* regions and a binary matrix is obtained for each subject with each entry in the matrix representing the existence or absence of a functional/effective connectivity between the corresponding two regions. The task now is to detect those links that appear with significantly different frequencies in patients and healthy controls.

For a particular link, assume it appears with probability *p* in healthy controls, and *q* in patients. The score *S* = *p* − *q* then represents the difference of the occurrence probabilities between the two groups of subjects. Let ρ_*H*_ and ρ_*P*_ be the proportions of the healthy controls and patients in a sample with this link. If the subjects are independent and the sample size is large, according to the Law of Large Numbers, *S* can be approximated by the difference of the proportions ρ_*H*_ − ρ_*P*_. Furthermore, let *L*_*H*_ and *L*_*P*_ denote the number of this link present in the individual networks of healthy controls and patients respectively. Then the independence assumption implies that *L*_*H*_ and *L*_*P*_ follow binomial distributions *B*(*N*_*P*_, *p*) and *B*(*N*_*H*_, *q*) respectively. Hence, if *N*_*H*_ and *N*_*P*_ are large and *p*, *q* are not close to 0 and 1, ρ_*H*_ and ρ_*P*_ are approximately normally distributed. More specifically, ρ_*H*_ ~ *N*(*p*, *p* (1 − *p*)/*N*_*H*_) and ρ_*P*_ ~ *N*(*q*, *q*(1 − *q*)/*N*_*P*_) where *N*(μ, σ^2^) is the normal distribution with mean μ and variance σ^2^. In the present study, we did not consider those links with both ρ_*H*_ and ρ_*P*_ smaller than 0.02 or larger than 0.98 to ensure the validity of this approximation and at the same time release the burden for multiple comparisons. Therefore,

$$\rho_{H}-\rho_{P}\sim \;N(p-q,p(1-p)/N_{H}+q(1-q)/N_{P}).$$

In practice, the score *S* is estimated from the data as

$$\hat{\text{S}}=\rho_{H}-\rho_{P}=L_{H}/N_{H}-L_{P}/N_{P}.$$

To assess how differential the link is between two groups of subjects, note that under the null hypothesis that no difference exists, we have *p* = *q* and the density of ρ_*H*_ − ρ_*P*_ is then centered at zero. Hence, the *p*-value for an observed score $\hat{\text{S}}$ can be calculated as $\Phi (-|\hat{S}|/\hat{\sigma })$, where $\Phi \left(\cdot \right)=\frac{1}{\sqrt{2\pi }}\int_{-\infty }^{\cdot }{\text{e}}^{{\text{t}}^{2}/2}\text{dt}$ is the cumulative distribution function of the standard normal distribution and ${\hat{\sigma }}^{2}={\hat{\rho }}_{H}(1-{\hat{\rho }}_{H})/N_{H}+{\hat{\rho }}_{P}(1-{\hat{\rho }}_{P})/N_{P}$. Equivalently, a threshold for $\text{|}\hat{\text{S}}\text{|}$ to claim α-level significance could be specified as ${\text{S}}_{\text{th}}=-\hat{\sigma }\Phi^{-1}(\alpha )$. It can be seen that an increase of population size will reduce the estimated variance ${\hat{\sigma }}^{2}$ and thus increase the power of the test.

Since the association is tested for a large number of links, a correction for *p*-values is needed to account for multiple comparisons. In the present study, a false discovery rate (FDR) procedure is used.

#### Feature integration

To reduce the dimension of each feature, we integrate each high dimensional feature by applying a dimensionality reduction scheme. For example, for partial correlation network (which is 4005 dimensional), we derive a two dimensional feature by grouping the links in the partial correlation network into two sets: one is composed of links that have a stronger correlation in ADHD patients than in health controls; and the other is composed of links with weaker correlation in ADHD patients (i.e., those links with positive and negative *t*-score of two-sample *t*-test imploded in feature selection, respectively). A two dimensional feature is then obtained by averaging the links in these two groups, respectively. The feature of spatial correlation is integrated in the same way. For Pearson correlation network, the principal component analysis (PCA) (Malhi and Gao, 2004) is used extract the most useful information. We select the first *m* principle components that leads to an *m*-dimensional feature (here we choose *m* = 8). Finally, to extract the key information contained in neuroimaging markers of fALFF and ReHo. Firstly, a multiple comparison is performed to avoid noise voxels. Then we obtain an averaged ReHo value using voxels that are significantly increased in the patient group, which are defined as mean increased ReHo. The mean decreased fALFF value can be acquired in the same way, which results in a two-dimensional feature for these two neuroimaging markers, respectively.

#### Classifier

**Figure 1:**
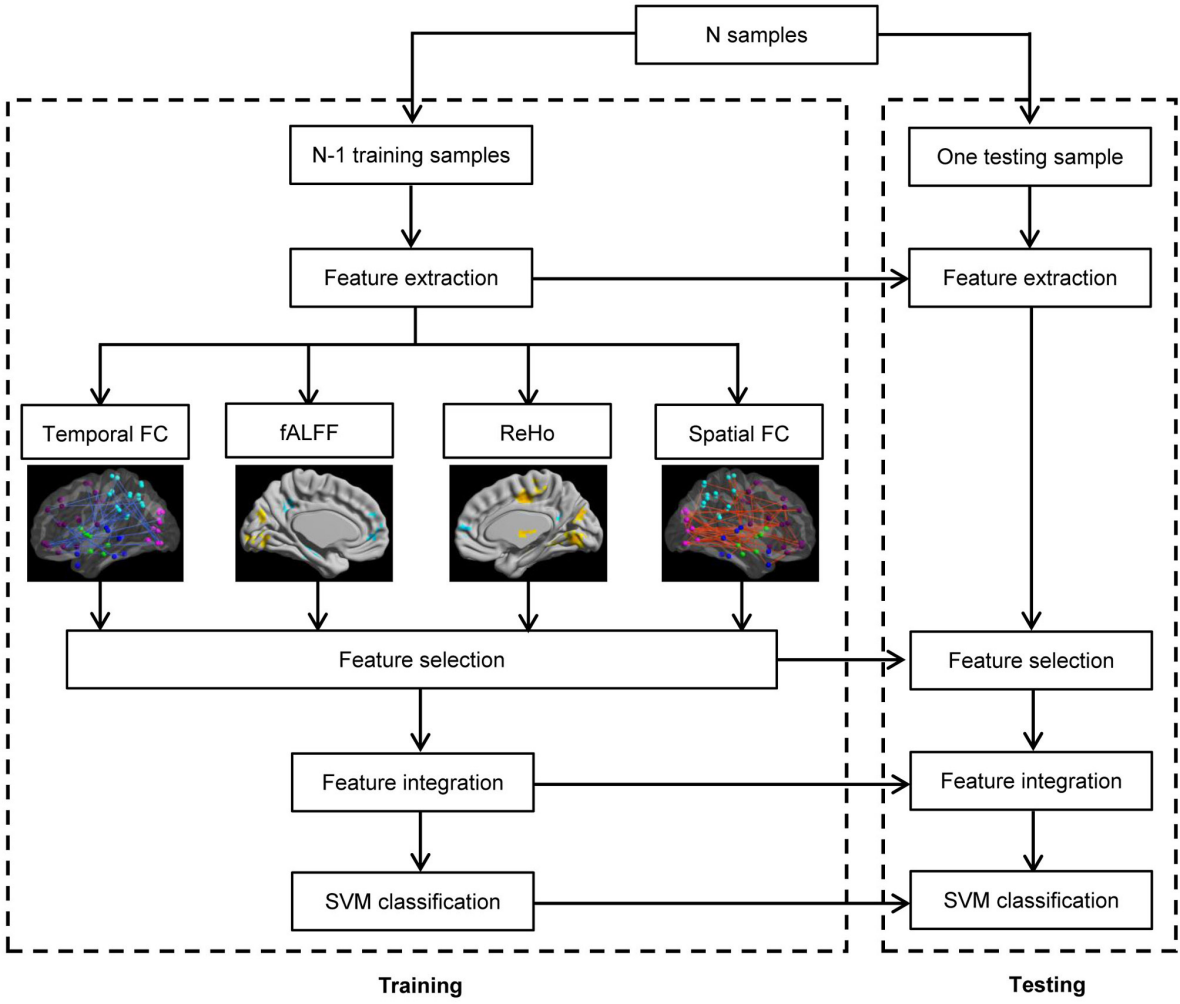
**Flowchart of the pattern recognition framework proposed**.

The framework of our proposed ADHD classification scheme is shown in Figure 1. The classifier adopted here is the support vector machine (SVM) implemented with libsvm version 3.1 (Chang and Lin, 2011), and the aforementioned features are used to perform the classification. We used Gaussian kernel in the SVM classifier. The kernel width *h* and the regularization parameter *C* in SVM are determined by standard fivefold cross validation implemented in libsvm software package. In the prediction part, we applied the leave-one-out cross-validation, i.e., we use a single subject from the original data as the test data (or validation data), and the remaining subjects as the training data. This is repeated such that each subject is used once as the test data. The accuracy of a classifier is defined as *corr*/*sum*, whereas corr denotes the number of correctly classified subjects, and sum denotes the number of total subjects. The sensitivity and specificity evaluate the performance of a classifier to identify positive and negative instances, respectively, and they are defined as below:

$$\text{Sensitivity}=\frac{true\;positives}{true\;positives+false\;negatives}\times 100\%$$

$$\text{Specificity}=\frac{true\;negatives}{false\;positives+true\;negatives}\times 100\%$$

## Results

### Altered functional connectivity

**Figure 2:**
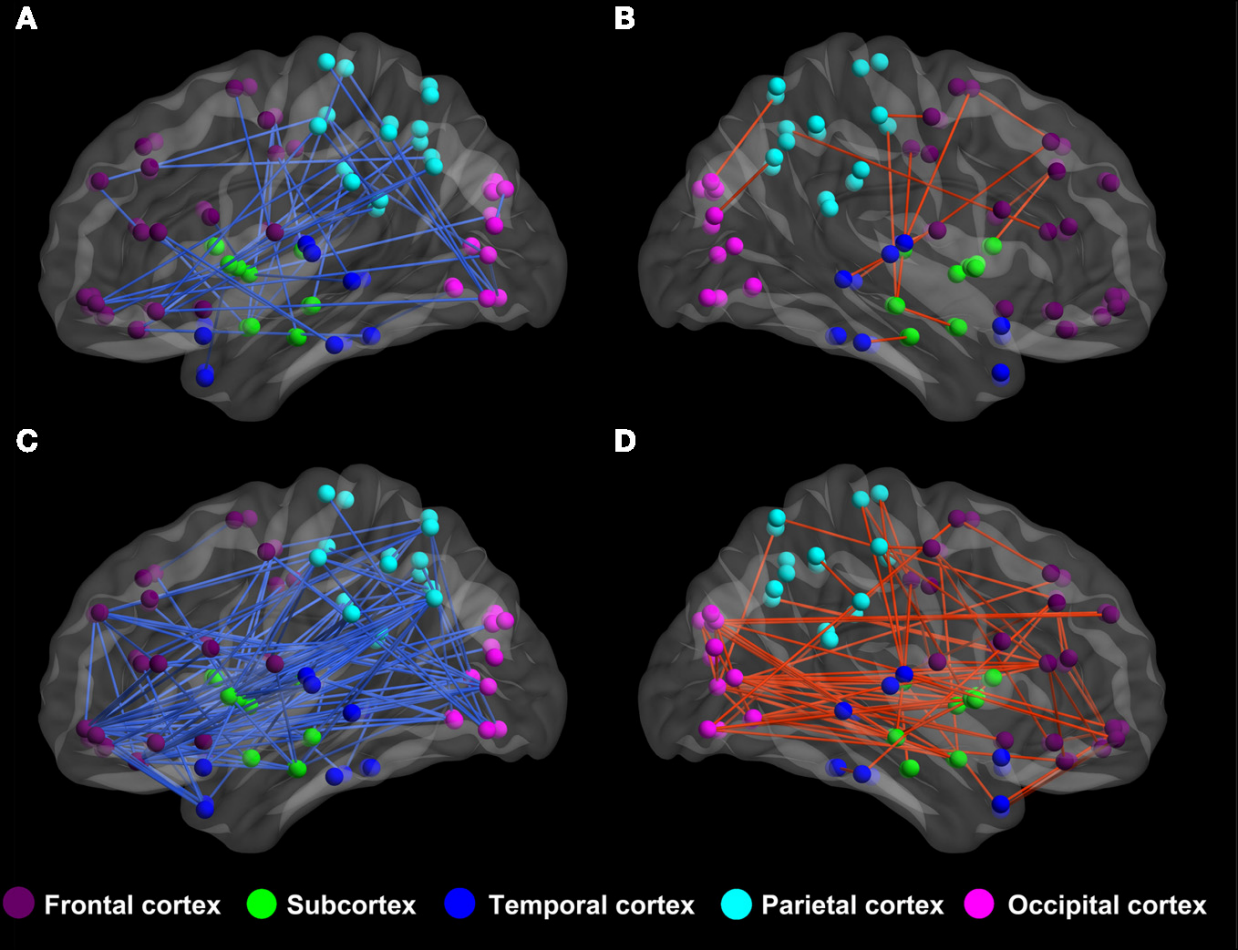
**The altered functional connectivity of ADHD patients compared to healthy controls. (A,B)** Altered functional connectivity in terms of partial correlation by BWAS approach (*p* < 0.008, which is also used in feature selection). Red lines represent significant links that appear more frequently in ADHD patients while the blue lines are links that appear more frequently in controls. **(C,D)** Altered functional connectivity in terms of spatial correlation (*p* < 0.008, which is also used in feature selection). Red lines represent those links that are increased in terms of spatial correlation in ADHD patients, while blue lines are links that show decreased spatial correlation in ADHD patients.

By applying BWAS method, the significantly changed functional connectivity in terms of the partial correlation are found to distribute mainly in the frontal lobe and the parietal cortex, see Figure 2. In the significantly altered 122 links (*p* < 0.008, which is also used in feature selection), 33.33% (37/122) links are associated with frontal cortex and 23.77% (29/122) links are associated with parietal cortex. It is worthy to note that among these links, the most significantly altered functional connectivity is the link between left hippocampus (HIP.L) and left amygdala (AMYG.L) (*p* = 2.4e-5) (Plessen et al., 2006). For the functional connectivity from spatial correlation, the altered links mainly distribute in the frontal cortex and subcortex. We found that 34.12% (216/633) and 23.85% (151/633) altered links are associated with these two areas, respectively. Both measures involve functional connections related to frontal cortex, which are consistent with previous findings using group comparison methods (Ashtari et al., 2005; Wang et al., 2009).

### fALFF and ReHo

**Figure 3:**
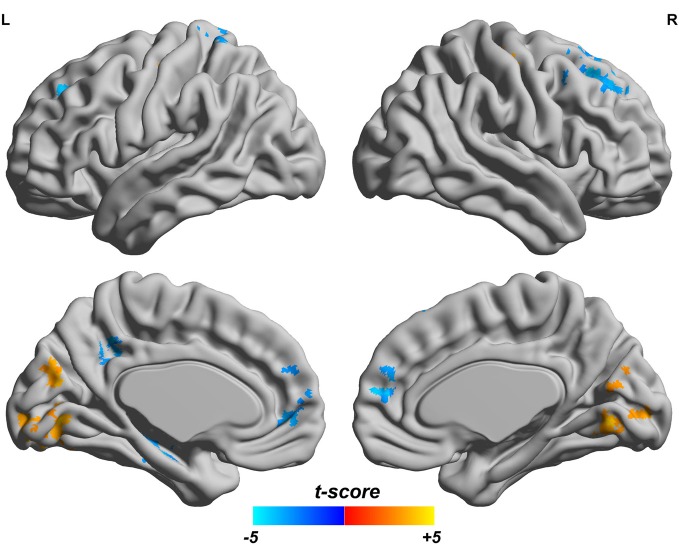
**Comparisons of fALFF between the ADHD patients and healthy controls.** T-score bar is shown at the bottom. Warm and cold colors indicate ADHD-related fALFF increase and decrease, respectively. Threshold is set at *p* < 0.05 (AlphaSim correction).

**Figure 4:**
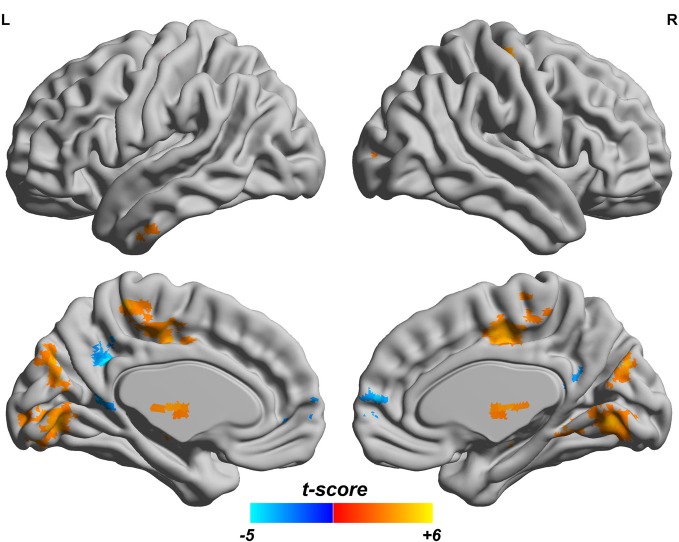
**ReHo difference map between the ADHD patients and healthy controls.** T-score bar is shown at the bottom. Warm and cold colors indicate ADHD-related ReHo increase and decrease, respectively. Threshold is set at *p* < 0.05 (AlphaSim correction).

Since both fALFF and ReHo are computed at the voxel level, we obtain two brain maps which reflect the abnormalities of “energy” of the voxels' activity and the local homogeneity in the ADHD patients, see Figures 3 and 4, respectively. Here a two-sample *t-test* is performed on voxel basis to spot the significantly different voxels across the two groups; the *t*-map of each group is corrected for multiple comparisons using the AlphaSim command in AFNI (Cox, 1996) and a corrected significance level of *p* < 0.05 is obtained by clusters with a minimum volume of 640 mm^3^ at an uncorrected individual voxel height threshold of *p* < 0.005. Compared with the healthy controls, the ADHD patients showed a significant fALFF increase in the bilateral lingual gyrus (LING), right precentral gyrus (PreCG.R) and left cuneus (CUN.L) and a decrease in the cerebellum, the bilateral superior frontal gyrus (SFG) and middle frontal gryus (MFG). Additionally, we also find many regions showing increased ReHo (labeled in warm color) in patient group, including the cerebellum, the bilateral LING, cuneus (CUN), Thalamus (THA), precentral gyrus (PreCG), and cingulate gyrus; while only a very small portion of voxels show a decrease in ReHo, including precuneus (PCUN) and medial frontal gyrus.

### Pattern classification results

**Figure 5:**
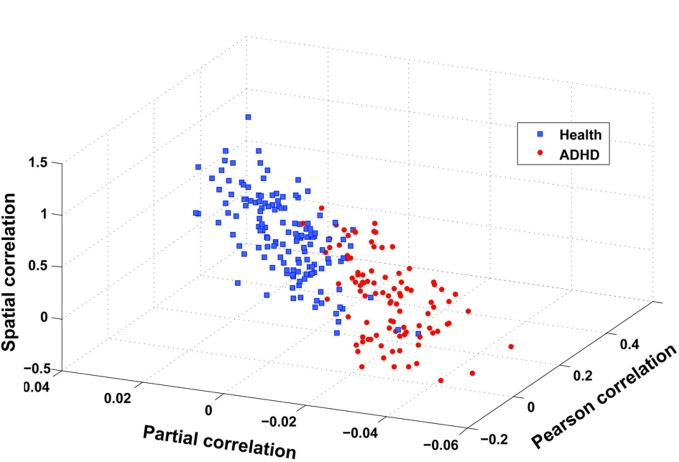
**Scatter plot of three features after integration, i.e., spatial correlation, partial correlation, and Pearson correlation (CC400).** These three features are obtained in the following way: taking spatial correlation for example, we calculate the mean of spatial correlation of those links that have a stronger and weaker correlation in ADHD patients than in health controls, respectively, and then take the difference between these two means.

**Figure 6:**
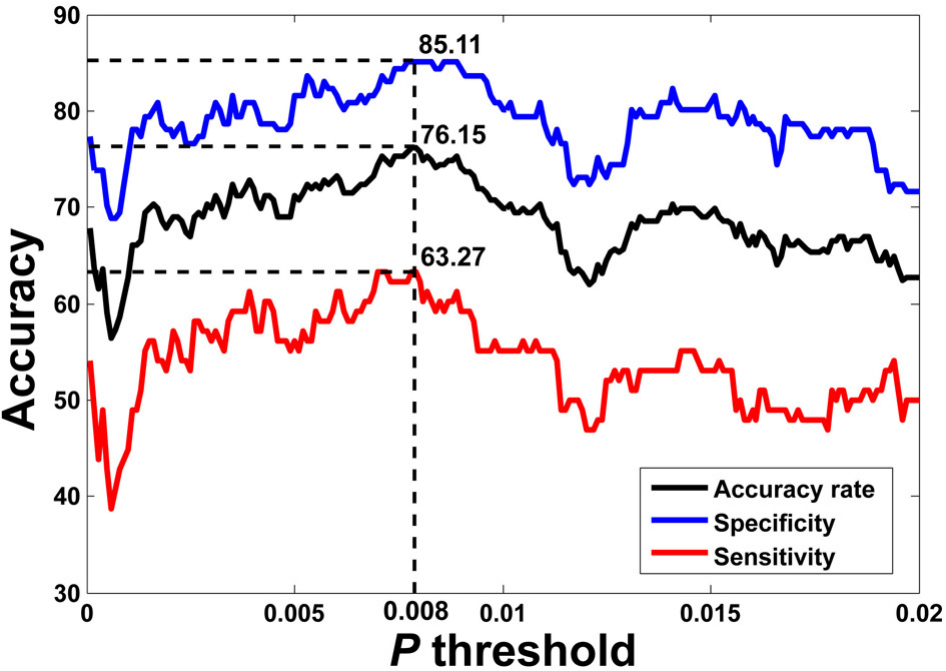
**LOOCV prediction accuracy of classifying ADHD patients from controls with respect to different *p*-value thresholds in feature selection.** Corresponding sensitivity and specificity are also plotted. It can be seen that for a wide range of *p*-value thresholds in selecting features for SVM, we can achieve more than 70% of accuracy in predictions. The best accuracy (76.15%) is achieved when *p*-value threshold is 0.008.

The flowchart of the pattern recognition framework is shown in Figure 1. The classification accuracy using this framework is listed in Table 2. We find that the features from Pearson and partial correlation of the functional connectivity perform the best in classification, reaching 70% of accuracy. The scatter plot using three kinds of functional connectivity (i.e., Pearson, partial and spatial correlation) is shown in Figure 5, from which we can see that the features from functional connectivity possess highly sensitive information to distinguish the healthy controls and ADHD patients. Other features, such as fALFF and ReHo are less sensitive, which lead to classification accuracy being about 65%. Since the features from distinct neuroimaging markers such as functional connectivity, fALFF, and ReHo represent functional organization of the brain from different aspects, a combination of all these features in pattern classification results in an overall accuracy of 76.15%. To test the robustness of our classification, we evaluate the classification performance with respect to different threshold (*p* value) used in features selection, with the results shown in Figure 6. It can be seen that for a wide range of thresholds adopted, we can achieve more than 70% accuracy in predictions. For *p*-value threshold being 0.008, the accuracy can reach 76%. Thus the features are more promising in classifying ADHD patients and controls. It should be noted in Table 2 that the specificity of our classification is higher that the sensitivity. The main reason is that the number of healthy controls (141) is larger than that of ADHD patients (98), which renders the hyper plane in SVM to be biased that favors the correct classification of healthy controls.

**Table 2:** **Classification results (LOOCV) using different imaging markers**.

**Metrics**	**Accuracy (%)**	**Sensitivity (%)**	**Specificity (%)**
All features	76.15	63.27	85.11
Partial correlation	71.13	56.12	81.56
CC400 Pearson correlation	67.78	46.94	82.27
Spatial correlation	63.18	40.82	78.72
Partial corr. +	73.22	58.16	83.69
CC400 corr. +			
Spatial corr.			
fALFF + ReHo	64.85	48.98	75.89

## Discussion

In this paper, we address the problem of accurately classifying individual state from a large dataset consisting of 141 healthy children and 98 ADHD patients. Using neuroimaging markers derived at different scales, such as fALFF, ReHo, and various kinds of functional connectivity measures, we have identified the most discriminative features for accurate classification on the individual level. The frontal and cerebellar regions are found to change significantly across the two groups, and the correlation between these neuroimaging markers and the ADHD index is also presented. Finally, the further improvement, i.e., a multi-modal approach is discussed to extend our results.

### Significantly changed brain regions in ADHD

ADHD is characterized by clinical symptoms of inattention, impulsivity, and hyperactivity, either alone or in combination. Many neuropsychologists believe the pathophysiology of this disorder may involve dysfunction of frontal–striatal–cerebellar circuits (Krain and Castellanos, 2006). Moreover, anatomical imaging studies among ADHD patients are consistently related to the frontal lobes, basal ganglia, corpus callosum, and cerebellum. (Jay N. Giedd, Brain Imaging of Attention Deficit/Hyperactivity Disorder). Summarizing our main results (i.e., significantly changed brain regions in ADHD patients) from other neuroimaging markers, we note that the frontal lobe and the cerebellum are among the most relevant regions underlie ADHD patients. The frontal cortex is known to be involved in multiple aspects such as planning, working memory, learning, and emotional regulation and it also modulates activity in subcortical structures like limbic areas, giving rise to the ability to engage in inhibitory control over behavior (Miller and D'Esposito, 2005; Marsh et al., 2008). Importantly, frontal lobe are thought to support selective and divided attention, attention shifting, and executive control, (Posner and Petersen, 1990; Duncan and Owen, 2000). Our finding of the altered functional connectivity of prefrontal cortex suggests that children with ADHD may be unable to recruit prefrontal regions for control of behavior, including the inhibition of hyperactivity and the precise motor control. This is consistent with previous work, which point out that the frontal and cerebellar region abnormalities may contribute to the pathophysiology of ADHD (Mulder et al., 2008; Mahone et al., 2011; Tomasi and Volkow, 2011). In terms of structural alteration of the frontal area, it has been demonstrated that the decrease in frontal lobe volume in ADHD accounted for 48% of the reduction in total cerebral volume (Mostofsky et al., 2002). For cerebellum, current neuropsychological findings implicate that it is not only related to locating motor movements but is also involved in non-motor behaviors such as timing and shifting attention through connections with frontal areas (Allen et al., 1997; Sobel et al., 1998; Tracy et al., 2000). All the above findings indicate the important roles of cerebellar and frontal lobe in ADHD. The significantly changed brain regions identified by our approach may be helpful in understanding the detailed pathophysiology of ADHD.

### Correlation between identified neuroimaging markers and ADHD index

**Figure 7:**
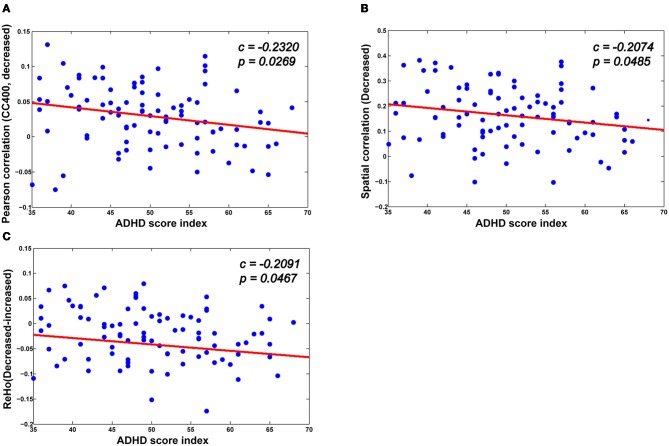
**The correlation between various biomarkers and ADHD indices. (A,B)** The mean decreased Pearson correlation and spatial correlation show negative correlation with the ADHD index. **(C)** There is also a negative correlation between the altered ReHo (the difference between the mean decreased ReHo and increased ReHo) and the ADHD index.

As can be seen in Figure 7, there is a significant association between the derivatives from various neuroimaging markers of the ADHD patients and the ADHD index. For the neuroimaging markers such as ReHo and the functional connections, we first identify the significantly changed voxels (for ReHo), and the edges (for the functional connections) using the above mentioned multiple hypotheses tests. We then divide each high-dimensional feature from the patients into two groups: those voxels or functional connections that are higher or lower than those of the healthy controls in terms of the neuroimaging marker used, respectively. For each group, we then calculate the mean value as an integration of the high-dimensional feature, which we find to correlate significantly with the ADHD index. From Figure 7, we can see that for those functional connections and ReHo that are decreased in the ADHD patients, there is a negative correlation between the sum of the decreased functional connections (also ReHo) and the ADHD index. These findings suggest the clinical relevance of the neuroimaging makers adopted in our study, indicating that the change in the neuroimaging markers in the patients group are closely related to the severity of ADHD.

### Towards an automatic classification of ADHD

In the current paper, we have developed a classifier which can accurately discriminate ADHD patients from healthy controls. Although we have achieved a relatively high accuracy of discrimination, we have much space for further improvements. With BOLD signals, we can include effective networks as further features (Zou et al., 2009; Luo et al., 2011). The structural MRI data, which should be informative is not included in the present study, such as the T1-weighted signals and other modalities such as diffusion tensor images etc. (Raichle, 2006; Zhang and Raichle, 2010). Furthermore, the information contained in genes and SNPs should also be valuable for our discrimination. In summary, to achieve a more reliable diagnosis of various brain disorders, we have to take up a multi-modal approach that is promising in accurate classification of brain disorders.

### Conflict of interest statement

The authors declare that the research is performed without any commercial or financial relationships that could be construed as a potential conflict of interest.
